# Estrogenic Activity of Coumestrol, DDT, and TCDD in Human Cervical Cancer Cells

**DOI:** 10.3390/ijerph7052045

**Published:** 2010-05-04

**Authors:** Kenneth Ndebele, Barbara Graham, Paul B. Tchounwou

**Affiliations:** 1 The Laboratory of Cancer Immunology, Target Identification and Validation, College of Science, Engineering and Technology, Jackson State University, 1400 Lynch Street, P.O. Box 18540, Jackson, MS 39217, USA; E-Mails: kenneth.ndebele@jsums.edu (K.N.); Barbara.e.graham@jsums.edu (B.G.); 2 Molecular Toxicology Research Laboratory, NIH- Center for Environmental Health, College of Science, Engineering and Technology, Jackson State University, 1400 Lynch Street, P.O. Box 18540, Jackson, MS 39217, USA

**Keywords:** xenoestrogens, Coumestrol, DDT, TCDD, Cell Cycle

## Abstract

Endogenous estrogens have dramatic and differential effects on classical endocrine organ and proliferation. Xenoestrogens are environmental estrogens that have endocrine impact, acting as both estrogen agonists and antagonists, but whose effects are not well characterized. In this investigation we sought to delineate effects of xenoestrogens. Using human cervical cancer cells (HeLa cells) as a model, the effects of representative xenoestrogens (Coumestrol-a phytoestrogen, tetrachlorodioxin (TCDD)-a herbicide and DDT-a pesticide) on proliferation, cell cycle, and apoptosis were examined. These xenoestrogens and estrogen inhibited the proliferation of Hela cells in a dose dependent manner from 20 to 120 nM suggesting, that 17-β-estrtadiol and xenoestrogens induced cytotoxic effects. Coumestrol produced accumulation of HeLa cells in G2/M phase, and subsequently induced apoptosis. Similar effects were observed in estrogen treated cells. These changes were associated with suppressed bcl-2 protein and augmented Cyclins A and D proteins. DDT and TCDD exposure did not induce apoptosis. These preliminary data taken together, suggest that xenoestrogens have direct, compound-specific effects on HeLa cells. This study further enhances our understanding of environmental modulation of cervical cancer.

## 1. Introduction

Endogenous estrogens, especially 17-β-estradiol, have significant impact on cell mediated and humoral immune and autoimmune responses [1–5]. Derived from plant or industrial synthesis, environmental xenobiotics with potential estrogenic or hormonal activities are known as xenoestrogens. These compounds are ubiquitous, exhibit bioaccumulation, and act as estrogen agonists or antagonists, disrupting normal endocrine axes [6–15]. Xenoestrogens have significantly weaker binding affinities than endogenous estrogens to traditional steroid receptors [9,14,15] and their medical, environmental, and societal impact is the frequent subject of debate [12,13]. Representative xenoestrogens include compounds such as coumestrol, a phytoestrogen found in high levels in legumes that acts as an estrogen agonist. Coumestrol has been shown to modulate production of thymic hormones [17]. DDT (o,p-dichlorodiphenyltrichloroethane), a synthetic organochlorine pesticide, has a weak estrogenic agonist activity (as well as androgen antagonist activity) [21] and has been associated with immunosuppression in murine models [22–24] and modulation of cell cycle and apoptosis [25–27], but its effects on other diseases such as cervical cancer has not been characterized. TCDD (tetrachlorodibenzo-p-dioxin), a polychlorinated biphenyl dioxin, has been widely studied with variable results, having both estrogen agonist and antagonist activity [6,14,21]. It has also been found to modulate cell cycle proteins [29], induce thymic involution [30], and modulate cytokine expression [31,32]. Xenoestrogens may act at the cellular and molecular levels, binding to both steroid and aryl hydrocarbon receptors exhibiting both dependent and independent receptor modulations of specific gene transcriptional elements [29,32–35]. As a result, xenoestrogens have the potential to variably modulate cell proliferation, cell cycle progression, apoptosis and cytokine production in much the same way as 17-β-estradiol does [36–40]. This modulation is likely to occur in association with alterations in bcl-2 or p53 protein levels [29,38,39]. In this investigation, using the HeLa cell line as a model we explored xenoestrogen-specific effects.

## 2. Material and Methods

### Reagents and Cell Culture

Human cervical cancer (HeLa) cells were purchased from American Type Culture Collection (Rockville, MD), maintained in logarithmic growth, and cultured in DMEM. Cells were cultured in a density between 0.1 and 1.0 × 10^6^/mL. Medium was deemed complete when supplemented with 10% fetal bovine serum, 2 mM L-glutamine, 100 U/mL penicillin and 100 μg/mL streptomycin. The cells were cultured in suspension at 37°C and 5% CO_2_ in a humidified incubator and carried at 0.1–2.0 × 10^6^ cells/mL, passaging two to three times weekly as needed. Cells were pelleted and resuspended in fresh complete medium in tissue culture plates 24 h before use in experiments to avoid any confounding gene expression that might occur because of handling. Xenoestrogens were dissolved in 1,4-dioxane or DMSO with final concentrations of solvent in control or treated cultures < 0.1%.

### Proliferation Assay

Cells were cultured in triplicate at 1.0 × 10^6^/mL for 72 h, treated with different concentrations (0, 20, 40, 60, 80, 90 nM) of 17-β-estradiol, coumestrol, DDT and TCDD. Proliferation was measured by determination of total viable cell mass using the CellTiter 96® AQueous Non-Radioactive Cell Proliferation Assay Kit (Promega, Madison, WI) according to the manufacturer’s instructions. Absorbance at 490 nm was determined on a Bio-Rad (Hercules, CA) plate reader.

### Cell Cycle Analysis

Cell cycle analysis was performed as previously described [38,39]. Briefly, 1 × 10^6^ cells /mL were grown in suspension, harvested by centrifugation, washed, and fixed in 1% paraformaldehyde. After washing, the cells were permeabilized in 70% ethanol, washed, and re-suspended in PBS. RNase (Sigma) was added at a final concentration of 5 U/mL. Cells were stained with propidium iodide (PI). Flow microfluorometry was performed and DNA histograms were generated and analyzed using a Becton Dickinson Flowscan (Franklin Lakes, NJ). This method correlates closely with other measures of apoptosis including TUNEL and Annexin V staining while providing additional cell cycle information [41]. This method also allows for enumeration of the percentages of cells in G0/G1 (resting phase), S (DNA synthesis phase), G2M (mitotic phase), and hypodiploid or apoptotic percentages (those cells containing less than the normal amount of DNA) [41].

### Western Blot Analysis

For Western analysis, 5 × 10^6^ cells were cultured, treated with various concentrations of estrogen, coumestrol, DDT and TCDD compounds. Total protein concentration was determined by the method of Bradford using Bio-Rad Protein Assay reagents (Bio-Rad) in a microtiter assay plate. Total cellular protein (30ug) was electrophoresed on 12.5% SDS-PAGE gel, transferred to a polyvinylidine difluoride membrane (Amersham, Arlington Heights, IL) by electroblotting overnight. Membranes were blocked with 10% electrophoresis grade biotin-depleted non-fat dry milk (BioRad) in 1 X PBS (10 mM Tris pH 7.5, 100 mM NaCl, 0.1% Tween-20), rinsed in PBS, probed with monoclonal mouse anti-human bcl-2, Cyclin A and D (BD Bioscience San Diego, CA) using 1:1,000 dilution, and washed 3 times in PBS. The secondary antibody was HRP-conjugated goat anti-mouse whole IgG used at 1:1,000 (Transduction Laboratories). All antibodies were diluted in 1% milk in TBS. Membranes were washed three times. Detection of membrane-bound proteins was carried out by enhanced chemiluminesence with an ECL reagent kit (using 0.06 mL/cm^2^ of reagent) and Hybond autoradiography film (both Amersham). Biotinylated standards were used for molecular weight determination and were detected with 1:3,000 streptavidin-horseradish peroxidase (Amersham).

## 3. Results

### Xenoestrogen Effects on HeLa Cell Proliferation

**Figure 1.**
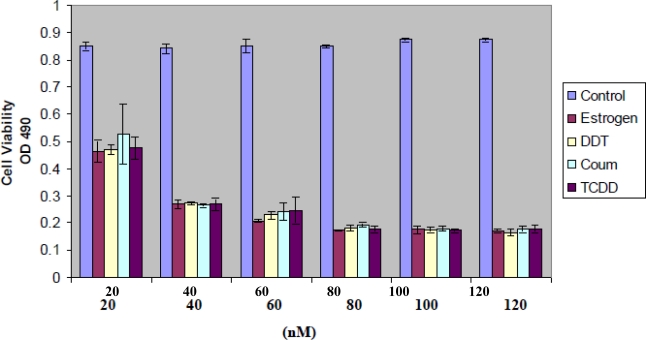
HeLa cell viability over 72 h exposure to 20 to 120nM 17-β-estradiol (E), DDT (D), Coumestrol, TCDD *vs*. control/solvents (C). 17-β-estradiol, DDT, Coumestrol and TCDD had profound effects on HeLa cell proliferation.

Study results show compound-specific effects on accumulation of viable HeLa cell mass. 17-β-estradiol and TCDD were used as representative controls of endogenous and environmental anti-estrogen compounds. In Figure 1, coumestrol, DDT and TCDD exhibited concentration dependent (20 to 120 nM) suppression on HeLa cell proliferation, indicating the cytotoxic effects of 17-β-estradiol [36–39]. Viable cell mass was assessed by MTT assay (see Methods). Change in total cell number was confirmed in cell cultures by enumeration (not shown). * *p <* 0.05 was determined using ANOVA with Bonferroni correction for multiple comparisons.

### Estrogen Suppresses Bcl-2 in a Dose-Dependent Manner

**Figure 2.**
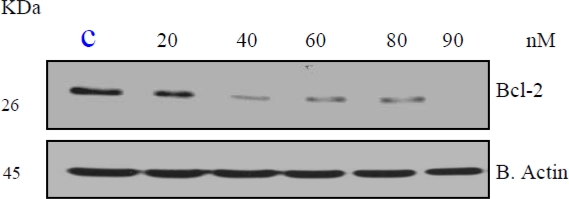
Expression of bcl-2 in HeLa cells exposed to17-β-estradiol (20, 40, 60, 80, and 90 nM) for 24 h. Western blot analysis of bcl-2 expression was performed as indicated in the Materials and Methods. Beta actin expression was used to assess equal lane loading.

Members of the bcl-2 family are crucial regulators of apoptosis in mammalian cells. The bcl-2 family includes antiapoptotic proteins, such as Bcl-2, and proapoptotic proteins, such as Bax. Since estrogen induced apoptosis is dependent on time and dose, we first tested bcl-2 response to estrogen. HeLa cells demonstrated a dose-dependent decrease (20, 40, 60, 80, 90 nM) in the expression of the antiapoptotic Bcl-2 protein upon estrogen treatment (Figure 2).

### Xenoestrogen Modulation of Cell Cycle Phase Distribution

**Figure 3.**
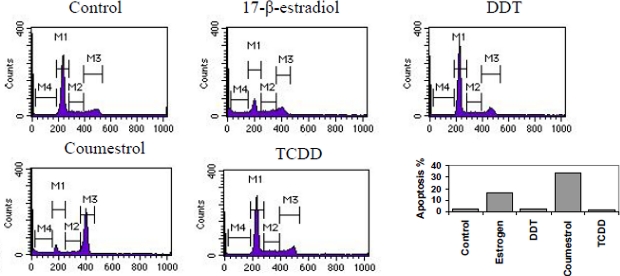
Histograms showing representative cell cycle phases of β-estradiol, coumestrol, DDT and TCDD treated HeLa cells for 24 H. Flow microfluorometry was performed as indicated in the Materials and Methods. Percent of apoptotic cells (labeled M4) is β-estradiol ∼17%, DDT ∼5%, Coumestrol shown for comparison. Control ∼0.2%, 17 ∼ 30% and TCDD ∼3%.

Cell accumulation or growth is a homeostatic balance between proliferation and apoptosis [42,43]. Questions have been raised regarding the MTT assay as a measure of viable cell mass, especially for xenoestrogens [44]. Therefore, xenoestrogen effects on cell cycle phase distribution in HeLa cells was also assessed by propidium iodide (PI) staining [38,39]. Representative cell cycle histograms for 17-β-estradiol, coumestrol, DDT and TCDD are shown in Figure 3, 17-β-estradiol and coumestrol had significant cell cycle phase effect on actively growing HeLa cells, causing redistribution from G0/G1 to apoptosis (*p <* 0.01), whereas TCDD and DDT had minimal effect, when analyzed by PI staining.

### Xenoestrogen Suppression of Bcl-2 and Stimulation of Cyclin A and D

**Figure 4.**
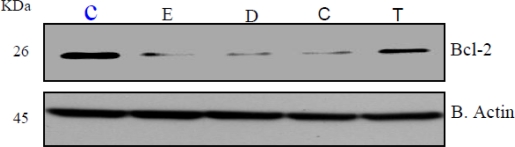
Expression of bcl-2 protein in β-estradiol, DDT, coumestrol, and TCDD treated HeLa cells for 24 h. Western blot analysis of bcl-2 expression was performed as indicated in the Materials and Methods. β actin expression was used to assess equal lane loading.

**Figure 5.**
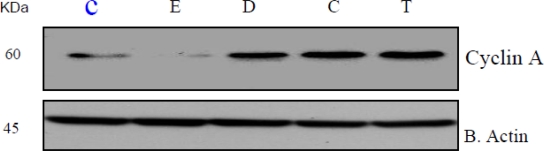
Expression of cyclin A protein in β-estradiol, DDT, coumestrol, and TCDD treated HeLa cells for 24 h. Western blot analysis of cyclin A expression was performed as indicated in the Materials and Methods. β actin expression was used to assess equal lane loading.

**Figure 6.**
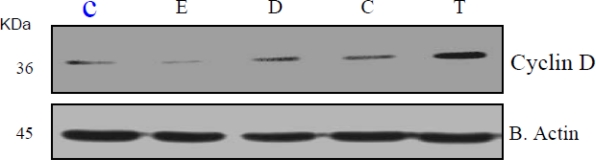
Expression of cyclin D protein in β-estradiol, DDT, coumestrol, and TCDD treated HeLa cells for 24 h. Western blot analysis of cyclin D expression was performed as indicated in the Materials and Methods. β actin expression was used to assess equal lane loading.

Bcl-2, Cyclin A and D cell regulatory proteins modulate cell cycle progression and apoptosis [42,43,46]. Given the observed effects of xenoestrogens on HeLa cell proliferation and cell cycle distribution, examination of bcl-2, Cyclin A and D protein levels was performed. As shown in representative Western blots (Figure 4; n = 3), xenoestrogens DDT, coumestrol, and 17-β-estradiol suppressed bcl-2 protein whereas, anti-estrogen, TCDD did not have a significant effect on bcl-2 expression as compared to the control. DDT, coumestrol and TCDD increased Cyclin A and D protein levels to variable degrees in HeLa cells (Figures 5 and 6 respectively). These preliminary results are consistent with recent reports of potential modulation of bcl-2 by xenoestrogens [47–50], supporting the concept that xenoestrogens may modulate cancer cell biology through associated changes in bcl-2.

## 4. Discussion

In this study we examined xenoestrogen’s direct actions on proliferation, cell cycle phase distribution and apoptosis in Hela cells. While the HeLa cells used in this study are transformed and may not accurately reflect primary cervical cells *in vivo*, they serve as a useful model and a basis for further examination of direct effects of xenoestrogens. As others have reported [51,52], and we confirmed in this investigation, xenoestrogens have a marked overall suppression effect on HeLa cell proliferation.

Xenoestrogen concentration used in this study, while supraphysiological, were comparable to those known to maximally stimulate estrogen receptor transcriptional activity [53]. Based on previous *in vitro* studies, xenoestrogens in the range of 20 nM to 120 nM demonstrate maximal effects on cell apoptosis and cell differentiation [28,54] and these high concentrations may be required in transformed cell lines. Although the concentrations in this investigation are some what higher that those found in the environment, long-term bioacccumulative actions may be anticipated to affect cancer cell biology. With receptor binding affinities up to 10,000 fold weaker than endogenous estrogens, the environmental impact of xenoestrogens is the subject of scientific and societal debate [51,55]. These preliminary studies of representative xenoestrogens have been designed to detect and dissect potential effects on HeLa cells.

Xenoestrogens are environmental hormones or compounds that exhibit estrogenic activity. They may interact with or disrupt endogenous estrogenic activity, and, as suggested by some investigations, may have implications for health and disease [27,56]. In the present study, estrogen, coumestrol and DDT, but not TCDD were shown to variably but significantly suppress bcl-2 protein expression in HeLa cells. However, DDT, coumestrol and TCDD upregulated cyclin A and D protein expression. The variability is likely due to xenoestrogenic potency with respect to estrogenic activity [57,58], as well as possible differences in mechanisms of action [23–25]. Nevertheless, our experimental data suggest that these specific xenoestrogens, at high concentrations, have specific effects on HeLa cells.

While concentrations of xenoestrogens used in this study may exceed those detected in the environment and the general population, chronic, low level of exposure is known to have biological effects [58,60]. The purpose of this investigation was to identify possible mechanisms of HeLa cell modulation and not necessarily establish environmental exposure-based cause-and-effect evidence. Furthermore, acute *in vitro* effects cannot be adequately extrapolated to chronic, low dose exposure *in vivo* effects. Hence, results in the current study should be interpreted with utmost caution. Observed effects occurred only in selected xenoestrogens, implying that effects may be compound-specific and that broad generalization for individual compounds are not appropriate. Data in the current study suggest a direct induction of apoptosis by estrogen and coumestrol, but not DDT AND TCDD. While verification and extension of our results is needed, the apoptosis induced in estrogen and coumestrol suggests at least one of potential effects, by which some xenoestrogens affect cell viability and induce cell death.

The effects observed in the current study may be estrogen receptor dependent or independent [61]. Jeon and Esser have shown that TCDD elicits its biological function through binding of the AHR to distal DNA motifs [62]. However, xenoestrogens may also have AHR receptor independent effects on hela cells and xenoestrogen mechanisms of action are likely pleiotropic [63]. Delineating xenoestrogen-mediated effects through the ER or AHR is pivotal to understanding molecular mechanisms of xenoestrogens, but is beyond the scope of this initial study.
